# Chinese Intelligence Prescription System improves prescription accuracy while decreasing labor and drug costs

**DOI:** 10.1186/s12913-023-09487-4

**Published:** 2023-05-22

**Authors:** Ting-Yu Huang, Wei-Te Huang, Yu-Chuan Lin, Hao-Hsiu Hung, Shi-Chen Ou, Chin-Wei Chang, Hung-En Lin, Ting-Yen Lin, Ching-Wen Chang, Hui-Chun Hung, Sheng-Teng Huang

**Affiliations:** 1 Department of Chinese Medicine, China Medical University Hospital, North District, No. 2, Yude Rd, Taichung, 40447 Taiwan; 2 School of Post-Baccalaureate Chinese Medicine, China Medical University, Taichung, Taiwan; 3 Graduate Institute of Network Learning Technology, National Central University, Taichung, Taiwan; 4 School of Chinese Medicine, China Medical University, Taichung, Taiwan; 5 Research Cancer Center for Traditional Chinese Medicine, Department of Medical Research, China Medical University Hospital, Taichung, Taiwan; 6 An-Nan Hospital, China Medical University, Tainan, Taiwan

**Keywords:** Traditional Chinese Medicine, Precise prescription, Dispensing time, Labor cost, Drug cost

## Abstract

**Background and Aim:**

The traditional method of taking Chinese Medicine involves creating a decoction by cooking medicinal Chinese herbs. However, this method has become less popular, being replaced by the more convenient method of consuming concentrated Chinese herbal extracts, which creates challenges related to the complexity of stacking multiple formulas.

**Methods:**

We developed the Chinese Intelligence Prescription System (CIPS) to simplify the prescription process. In this study, we used data from our institutions pharmacy to calculate the number of reductions, average dispensing time, and resulting cost savings.

**Results:**

The mean number of prescriptions was reduced from 8.19 ± 3.65 to 7.37 ± 3.34 ($$p=2.46\;\times10^{-8}$$). The reduction in the number of prescriptions directly resulted in decreased dispensing time, reducing it from 1.79 ± 0.25 to 1.63 ± 0.66 min ($$p=1.88\;\times10^{-14}$$). The reduced dispensing time totaled 3.75 h per month per pharmacist, equivalent to an annual labor cost savings of $15,488 NTD per pharmacist. In addition, drug loss was reduced during the prescription process, with a mean savings of $4,517 NTD per year. The combined savings adds up to a not insignificant $20,005 NTD per year per pharmacist. When taking all TCM clinics/hospitals in Taiwan into account, the total annual savings would be $77 million NTD.

**Conclusion:**

CIPS assists clinicians and pharmacists to formulate precise prescriptions in a clinical setting to simplify the dispensing process while reducing medical resource waste and labor costs.

**Supplementary Information:**

The online version contains supplementary material available at 10.1186/s12913-023-09487-4.

## Introduction

Traditional Chinese Medicine (TCM) has been practiced for thousands of years. It focuses on treating patients based on regulating the internal balance of the human body [1]. Statistics from the Ministry of Health and Welfare (MOHW) of Taiwan showed that there were TCM departments in 133 hospitals and a total of 3,996 TCM clinics as of August 2021 [2]. Statistics from Taiwan note that almost 30% of the general population have received TCM therapy and consultations [3].

There are two primary methods by which Chinese herbal medicine (CHM) is prescribed in Taiwan. The first involves the traditional decoction method with medicinal Chinese herbs, while the second involves concentrated Chinese herbal extracts (CCHE) in granular form manufactured by GMP pharmaceutical companies. With regards to the second method, a single Chinese herb (single medicine) or whole decoction which contains multiple herbs (compound formula) are cooked based on descriptions and ratios recorded in ancient texts. The resulting water extract is mixed with excipients and subsequently dried to form powder-like granules known as concentrated extracts. Thus, there may be differences in terms of the composition and concentration ratios between different pharmaceutical companies.

Contemporary lifestyles dictate that the traditional method of preparing a decoction is generally unappealing and inconvenient due to the time and effort involved [4]. By comparison, CCHE is relatively convenient and acceptable to a population which is accustomed to processed and pre-packaged foods and medicines. Therefore, the use of medicinal decoctions has decreased significantly in recent decades, replaced by CCHE as the primary method of consuming TCM prescriptions in Taiwan [5]. Of note, during their medical training, TCM physicians are taught how to prepare a prescription based on the methods found in ancient texts, which are inconsistent with the CCHE prescription methods primarily used in clinical services.

Generally, TCM practitioners prescribe a combination of single herbals and compound formulas containing various dosages of CCHE. When stacking multiple compound formulas and single medicines together, it will become practically impossible for physicians to keep track of the actual dosage of every single herb contained within the prescription. Additionally, a specific herb may be unintentionally included with redundancy within the entire prescription. Several major problems are related to the prescription of CCHE. First, the dispensing of drugs by pharmacists often requires excessive effort and time due to redundancies and inefficiencies. Second, as a portion of the CCHE powder is lost during dispensing process, excessive herbal medicines and compound formulas are commonly used to create a prescription. Third, as physicians are unable to track the precise dosage of each herb, prescriptions containing excessive herbal components with potentially harmful or toxic effects may result.

Due to the abovementioned challenges, we designed the Chinese Intelligence Prescription System (CIPS) [Taiwan Utility Model Patent M617562]. The novel system takes into account the accurate composition of each single herb in compound formulas and uses an algorithm to calculate the number of single herb/compound formulas in each prescription [6]. In this study, we applied CIPS to analyze real-world data collected from the Chinese Medicine Department pharmacy at China Medical University Hospital (CMUH), to demonstrate a precise prescription and reveal the associated time and labor savings as well as reduced CCHE loss.

## Materials and methods

### Summary of the process of dispensing and estimation of minimal required time

To give readers an overall idea of the process of dispensing TCM drugs, the steps of dispensing each prescription are summarized below.

- Step 1. The pharmacist scans his/her barcode to initiate the counter to calculate the dispensing time.
- Step 2. The barcode on the prescription sheet is scanned.
- Step 3. The pharmacist retrieves a bottle from the shelf.
- Step 4. The barcode on the bottle is scanned into the computer which is connected to a digital scale.
- Step 5. The pharmacist removes the cap from the bottle. When a new bottle is used, the pharmacist must tear off a safety membrane.
- Step 6. The desired amount of powder is poured from the bottle into a bowl on the scale. A small quantity of powder is lost in this process as the fine particles spray into the air.
- Step 7. The barcode on the bottle is scanned again. If the reading from the scale is over the range of tolerance (difference between measured and prescribed values), a warning is issued, and the pharmacist will have to add or remove powder until the weight is within range.
- Step 8. The cap is screwed back onto the bottle.
- Step 9. The bottle is shelved.
- Step 10. The powder in the bowl is poured into a flask which will eventually contain the entire prescription. This is often done simultaneously with step 9. Again, some powder is lost when transferring.
- Steps 3 to 10 are repeated until all single herbs and compound formulas are retrieved. When the last bottle is scanned for the second time, the timer ends.
- The flask which now contains all prescription ingredients is attached to a mixer. The mixed powder will then be packaged for use by patients.

A demonstration of the process is shown in Supplementary Video 1.

According to the above steps, the minimum time required for dispensing a prescription is directly related to the number of bottles used. The least amount of time for bottle retrieval and opening is approximately 2 s, while a similar amount of time is required for capping and re-shelving. Thus, each bottle-related operation will take a minimum of 4 s. We consider this a minimum since it does not account for factors such as the process of pouring the powder into the bowl, taking bottles from distant shelves, and reaching for new bottles. If the prescription requires 7 bottles, the least amount of time required for dispensing would be 30 s; while 8 to 15 bottles would require at least 1 min; and 16 to 22 bottles would require at least 1.5 min. Data points incompatible with this stringent limit were excluded for this study.

### Example of stacking multiple compound formulas

As previously stated, TCM practitioners commonly prescribe a combination of single herbs and compound formulas, often resulting in unintended herbal redundancies. Here, we present a simple example of a prescription which only contains four compound formulas to demonstrate the occurrence of redundancies. In the given example, the physician prescribes Minor Center-Fortifying Decoction, Cinnamon Twig and Aconite Decoction, True Warrior Decoction, and Right-Restoring Pill. The compositions of these four compound formulas are listed in Table 1. If redundancies are included, these four compound formulas contain a total of 26 single herbs. However, one should note that Cinnamomum cassia, Paeonia lactiflora, Glycyrrhiza uralensis, Ziziphus jujuba appear twice, while Zingiber officinale, Aconitum carmichaelii appear thrice. Thus, the total number of single herbs in this prescription is actually only 18.

**Table 1:** An example of redundant herbs stacked together in four compound formulas. The single herbs are contained in the compound formulas, including Minor Center-Fortifying Decoction, Cinnamon Twig and Aconite Decoction, True Warrior Decoction, and Right-Restoring (Life Gate) Pill. The number of occurrences of each single herb present within these four compound formulas is also listed

Compound formula	Minor Center-Fortifying Decoction	Cinnamon Twig and Aconite Decoction	True Warrior Decoction	Right-Restoring (Life Gate) Pill	All single herbs used *Latin name*	Occurrences
Composition of each compound formula	*Cinnamomum cassia*	*Cinnamomum cassia*			*Cinnamomum cassia*	2
	*Paeonia lactiflora*		*Paeonia lactiflora*		*Paeonia lactiflora*	2
	*Glycyrrhiza uralensis*	*Glycyrrhiza uralensis*			*Glycyrrhiza uralensis*	2
	*Zingiber officinale*	*Zingiber officinale*	*Zingiber officinale*		*Zingiber officinale*	3
	*Ziziphus jujuba*	*Ziziphus jujuba*			*Ziziphus jujuba*	2
	*Maltose*				Maltose	1
		*Aconitum carmichaelii*	*Aconitum carmichaelii*	*Aconitum carmichaelii*	*Aconitum carmichaelii*	3
			*Wolfiporia extensa*		*Wolfiporia extensa*	1
			*Atractylodes macrocephala*		*Atractylodes macrocephala*	1
				*Rehmannia glutinosa*	*Rehmannia glutinosa*	1
				*Dioscorea polystachya*	*Dioscorea polystachya*	1
				*Lycium chinense*	*Lycium chinense*	1
				*Cuscuta chinensis*	*Cuscuta chinensis*	1
				*Eucommia ulmoides*	*Eucommia ulmoides*	1
				*Cervi Cornus Gelatinum*	*Cervi Cornus Gelatinum*	1
				*Cornus officinalis*	*Cornus officinalis*	1
				*Angelica sinensis*	*Angelica sinensis*	1
				*Cinnamomum cassia*	*Cinnamomum cassia*	1

The given example is of a prescription commonly issued by TCM practitioners. In an actual clinical setting, it can be challenging for physicians to keep track of the exact herbal components within each prescription. We thus developed an algorithm and the associated proprietary program (CIPS) to assist TCM physicians by simplifying the prescription process. The simplified procedure is described below.

### Simplified procedure

With CIPS [6], we have developed a program to identify and accurately calculate the components of TCM prescriptions. An example of the simplified steps performed by CIPS is described below. See our patent for details [6].

**Fig. 1:**
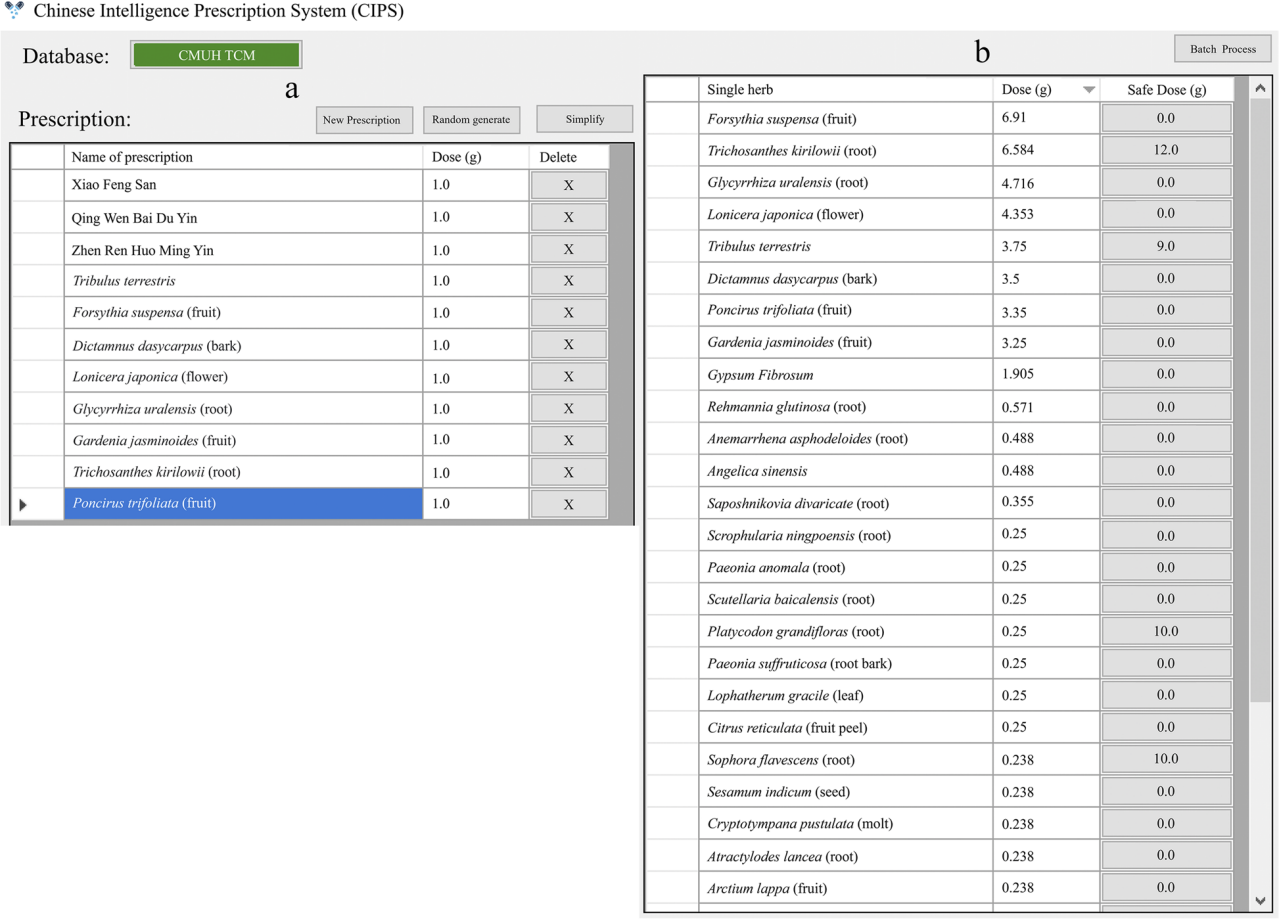
Original prescription (**a**) and single herbs (**b**) after unpacking the compound formulas. Note that the right panel is not the complete list due to screen limitations

Step 1. Single herbs and compound formulas are entered into the system (Fig. 1a). This example includes 11 single herbs and compound formulas. For simplicity purposes, we use the term “items” to include the sum of single herbs and compound formulas.

Step 2. The program unpacks the compound formulas to identify each single herb component. For example, Xiao Feng San is composed of the single herbs *Glycyrrhiza uralensis* (root), *Gypsum Fibrosum*, Rehmannia glutinosa (root), *Anemarrhena asphodeloides* (root), *Angelica sinensis*, *Saposhnikovia divaricate* (root), *Sophora flavescens* (root), *Sesamum indicum* (seed), *Cryptotympana pustulata* (molt), *Atractylodes lancea* (root), *Arctium lappa* (fruit), *Schizonepeta tenuifolia*. These single herbs are listed accordingly in Fig. 1b (due to the length of the list, *Schizonepeta tenuifolia* was omitted). Simultaneously, the total dose of each single herb is calculated. For example, 1 g of Xiao Feng San was decocted from 1.905 g of *Gypsum Fibrosum* and 0.571 g of *Rehmannia glutinosa* (root). These are also shown in Fig. 1b under the ‘Dose’ column.

**Fig. 2:**
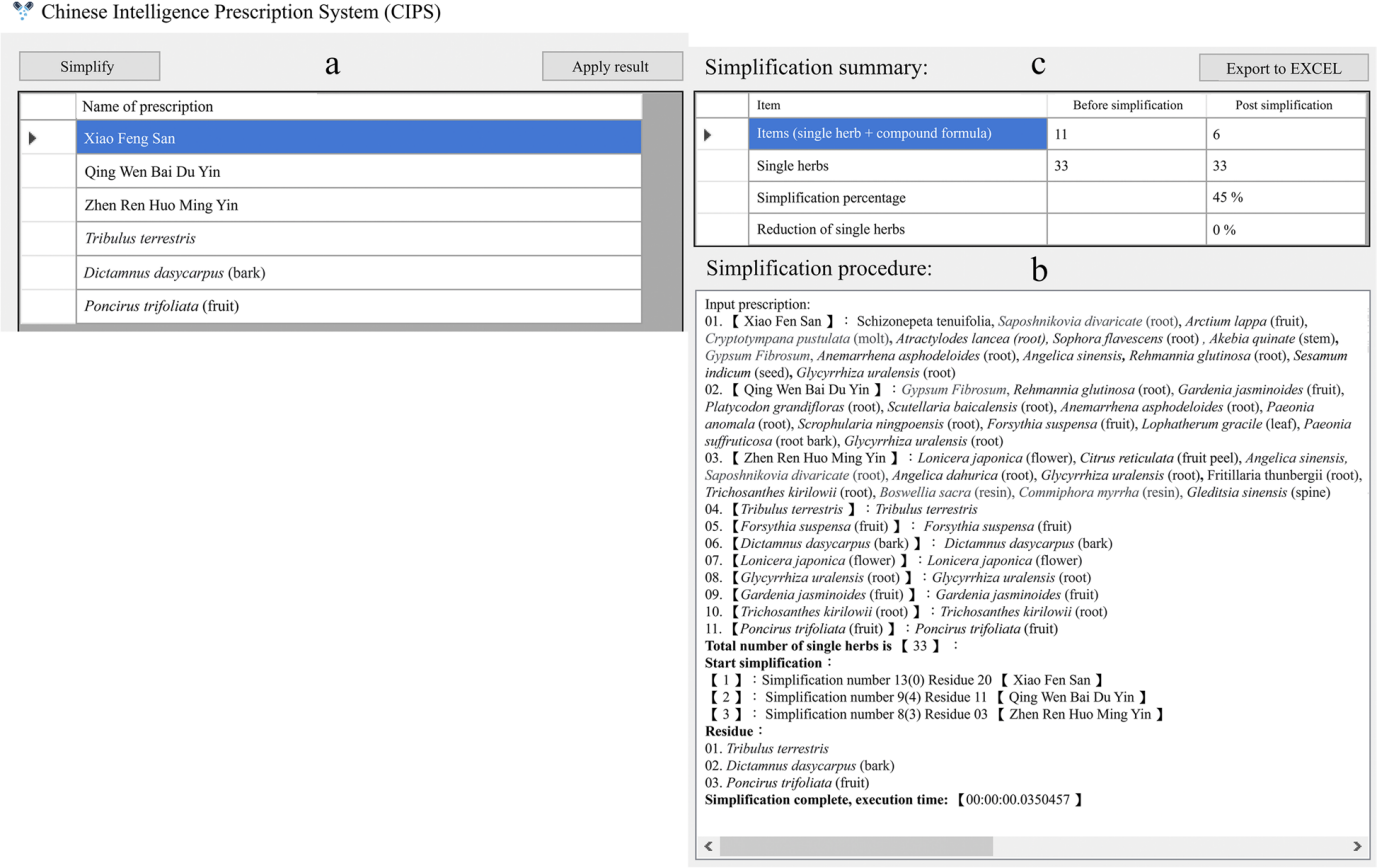
Prescription after algorithm (**a**), reduction procedure (**b**), and summary of items before and after simplification (**c**)

Step 3. Upon clicking “Simplify”, the program performs a simplification procedure whereby the algorithm analyzes all usable compound formulas in the database to select the best combination while including the least number of items. A detailed description of the simplification algorithm can be found in our patent details [Taiwan Utility Model Patent M617562]. As shown in Fig. 2a, the original prescription containing 11 items is subsequently simplified to include only 6. The reduction process and a comparison summary are shown in Fig. 2b and c, respectively. Since each hospital or clinic may acquire different pharmaceutical items have multiple suppliers, the program can be set according to each specific case.

A demo web version of our program is provided at http://122.116.18.16:8585/democmuh. Users are provided free access up to 5 times per day.

### Data processing workflow

**Fig. 3:**
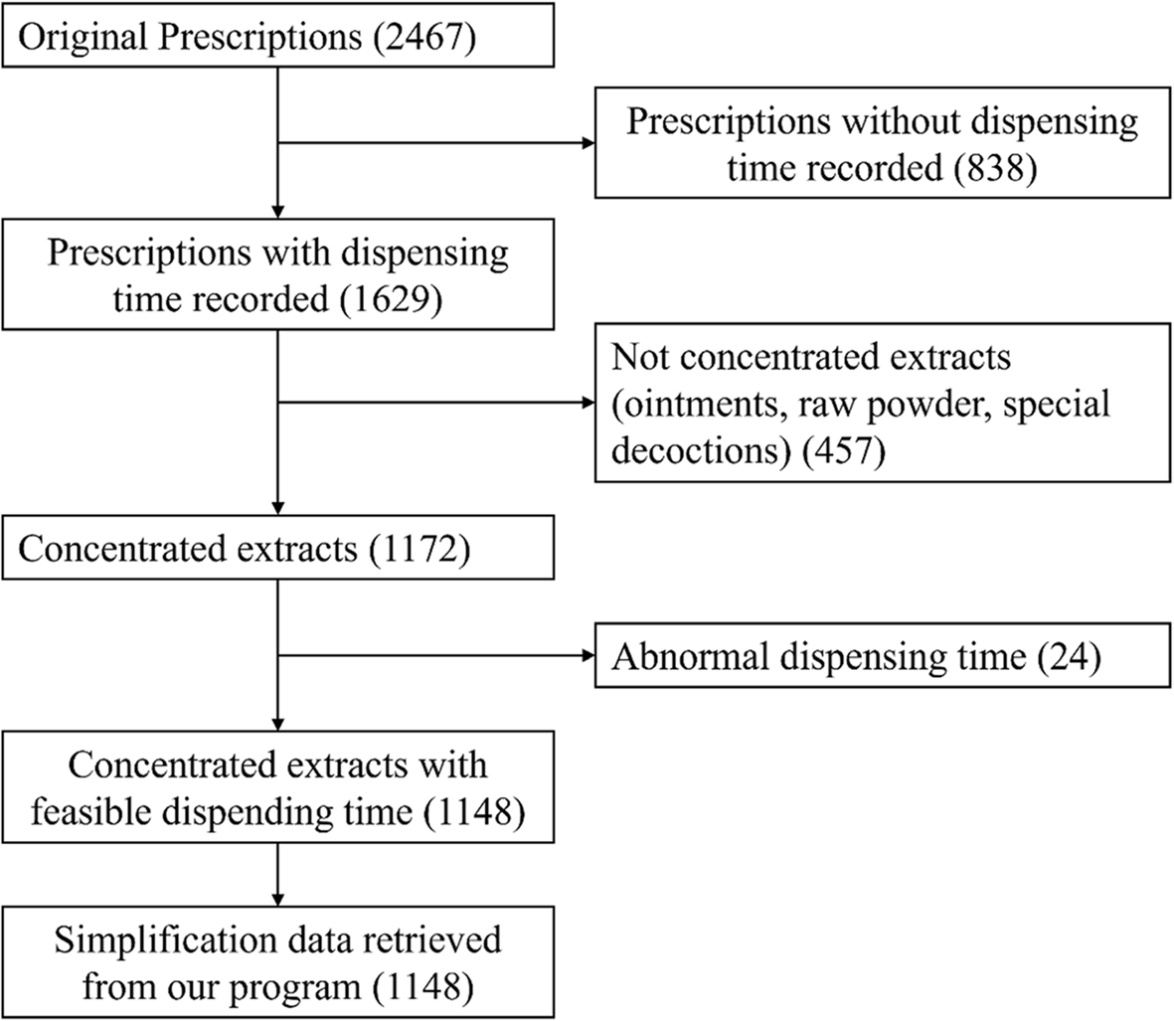
Flowchart of the prescription inclusion process

Prescription data from May 2019 of a representative pharmacist was obtained from our pharmacy. Original data included 2,467 prescriptions. Of these, only 1,629 prescriptions had the dispensing time recorded, the remaining 838 without dispensing time were excluded. In addition, 457 prescriptions did not include CCHE (ointments, raw powder, special decoctions) and were excluded, resulting in 1,172 prescriptions. Of note, 24 items did not meet our minimum dispensing time limit and were excluded, thus a total of 1,148 prescriptions were recruited for this study. The 1,148 prescriptions were entered into our program to arrive at the simplified results. A flowchart of the inclusion process is shown in Fig. 3.

### Powder spray loss estimation

To account for powder loss during the dispensing process, we estimated the mean loss by using a vacuum suction tube to remove powder from the surrounding surfaces. The weight of the collected powder was divided over the number of prescription items dispensed. The estimated mean loss was 0.13 g per item.

### Statistical analysis

The data we collected for this study consisted of continuous variables that passed the Kolmogorov–Smirnov test for normality. We propagated errors using standard error propagation methods. The significant criterion was set at < 0.05 for two-sided testing of a *p*-value.

## Results

### Time required for dispensing prescribed items

**Fig. 4:**
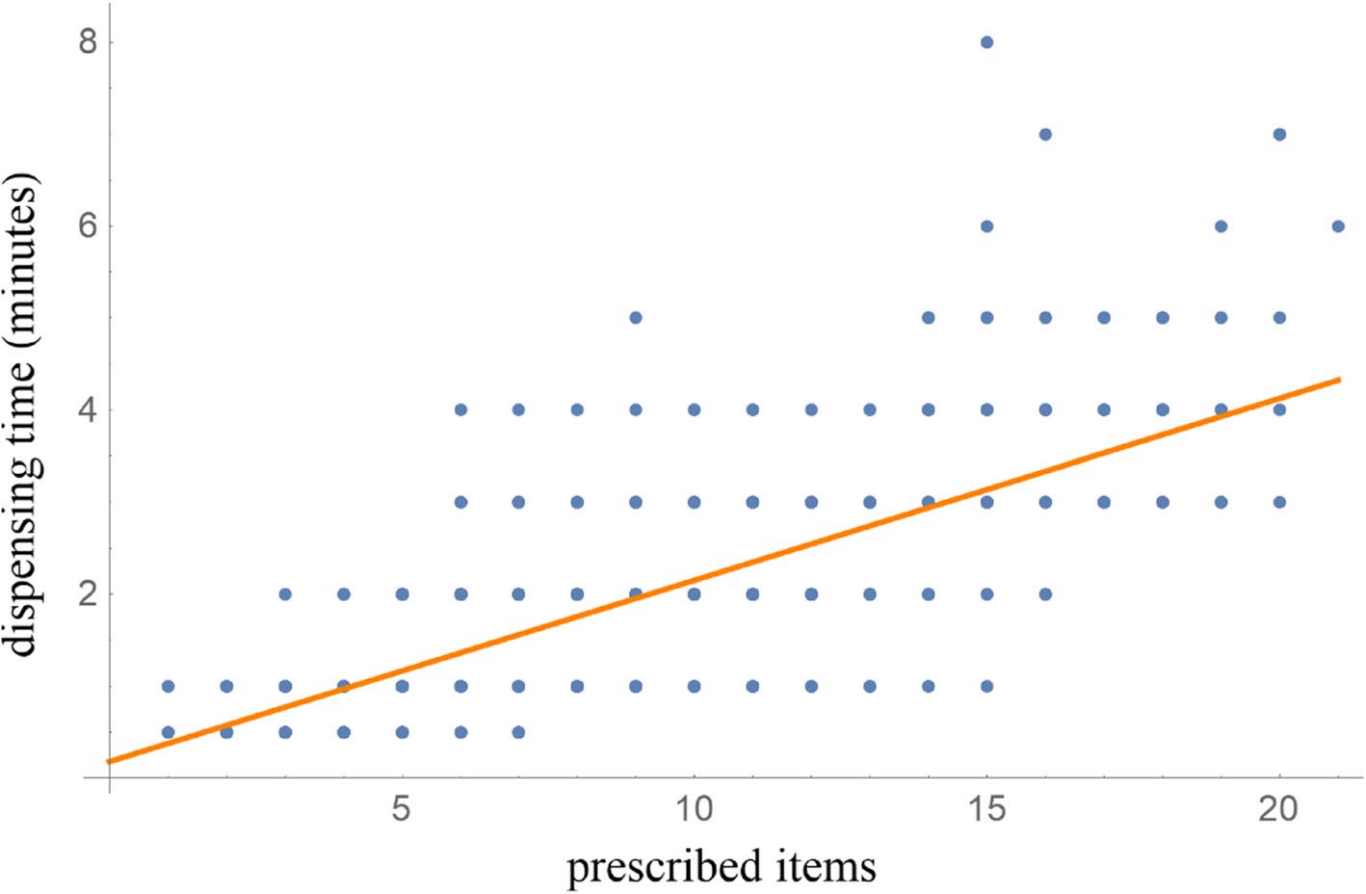
Calculation of prescribed items (X-axis) vs dispensing time (Y-axis, minutes)

The time needed for dispensing various items is shown in Fig. 4, which generally follows a linear trend. We calculated the trend-line as time = 0.1973 ($\pm 0.005$) x items + 0.1778 ($\pm 0.045$).

### Reduction of prescription items by applying the CIPS algorithm

The average total number of original prescription items including single herbs and compound formulas was $8.19\pm 3.65$. After applying the CIPS algorithm, the number of items was reduced to $7.37\pm 3.34$. The mean reduction was $0.82\pm 1.01$ items ($p=2.46\times10^{-8}$), translating into a reduction rate (number of items reduced divided by the original number of items) of $9.31\pm 11.27\%$. Detailed results regarding the different original numbers of items are demonstrated in Table 2.

**Table 2:** Prescription items before and after applying the CIPS algorithm

Prescription items (original) (A)	Number of dispensed items (B)	Prescription items (after calculation) (C)	Prescription items reduced (difference) (D)	*p*-value (E)	95% CI (F)	Percentage of reduction (G)
1	5	1	0	-	-	0
2	17	1.94 ± 0.24	0.06 ± 0.24	3.17E-01	(-0.06, 0.18)	2.94 ± 12.13
3	52	2.9 ± 0.3	0.1 ± 0.3	1.98E-02	(0.02, 0.18)	3.17 ± 9.82
4	81	3.72 ± 0.53	0.28 ± 0.53	8.51E-06	(0.16, 0.4)	7.1 ± 13.25
5	108	4.58 ± 0.61	0.42 ± 0.61	1.05E-10	(0.3, 0.54)	8.33 ± 12.27
6	129	5.52 ± 0.72	0.48 ± 0.72	6.22E-12	(0.35, 0.61)	8.05 ± 11.97
7	171	6.4 ± 0.72	0.6 ± 0.72	2.47E-21	(0.49, 0.71)	8.53 ± 10.24
8	140	7.2 ± 0.9	0.8 ± 0.9	2.00E-19	(0.65, 0.95)	10.21 ± 11.35
9	108	7.9 ± 1.13	1.1 ± 1.13	2.53E-17	(0.88, 1.32)	12.12 ± 12.4
10	89	8.9 ± 1.07	1.1 ± 1.07	1.34E-15	(0.87, 1.33)	11.01 ± 10.66
11	80	9.64 ± 1.14	1.36 ± 1.14	4.85E-17	(1.11, 1.61)	12.26 ± 10.25
12	43	10.42 ± 1.35	1.58 ± 1.35	1.38E-09	(1.16, 2.00)	13.21 ± 11.33
13	19	11.79 ± 1.18	1.21 ± 1.18	2.63E-04	(0.64, 1.78)	9.37 ± 9.05
14	22	12.64 ± 1.22	1.36 ± 1.22	3.03E-05	(0.82, 1.9)	9.55 ± 8.52
15	17	13.71 ± 1.21	1.29 ± 1.21	3.95E-04	(0.67, 1.91)	8.59 ± 8.05
16	16	14 ± 1.26	2 ± 1.26	9.65E-06	(1.33, 2.67)	12.63 ± 8.02
17	16	15.5 ± 1.26	1.5 ± 1.26	2.12E-04	(0.83, 2.17)	9 ± 7.59
18	18	16 ± 1.03	2 ± 1.03	1.61E-07	(1.49, 2.51)	11.28 ± 5.78
19	13	17.31 ± 1.55	1.69 ± 1.55	1.72E-03	(0.75, 2.63)	8.92 ± 8.22
20	4	18.25 ± 1.26	1.75 ± 1.26	4.99E-02	(-0.25, 3.75)	8.75 ± 6.29
8.19 ± 3.65	1148	7.37 ± 3.34	0.82 ± 1.01	2.46E-08	(0.76, 0.88)	9.31 ± 11.27

The number of original prescription items (A) with corresponding number of times dispensed by the pharmacist during May 2019 (B), prescription items left after applying the algorithm (C), number of items reduced (D), *p*-value for items reduced (E), 95% confidence interval of items reduced (F), the reduction percentage (G) for each of the original numbers of prescription items. The last row shows values for the average of all 1,148 dispensed items.

### Time saved by using the CIPS algorithm

Based on the above results, we calculated the time saved by reducing the number of items dispensed. Data from Table 2 was input to our linear regression model (Fig. 4) to calculate the time required for dispensing after the reduction, the time saved for each prescription, and the total time saved (Table 3). The average total number of original prescription items, including single herbs and compound formulas, was $8.19\pm 3.65$. The original mean dispensing time based on the linear fit model was $1.79\pm 0.25$ minutes. The estimated mean dispensing time after reducing the number of items was $1.63\pm 0.66$ minutes. The mean time saved during each dispensing was $0.16\pm 0.71$ minutes. When multiplied by the total number of dispenses, the total time saved from the item reduction process was 185 minutes. This indicates that CIPS would have saved the pharmacist approximately 3 h out of an average of 160 working hours each month.

**Table 3:** The prescription items associated with dispensing time saved by CIPS

Prescription items (original) (A)	Number of dispensed items (B)	Dispensing time (original) (C)	Dispensing time (reduced) (D)	Dispensing time (difference) (E)	*p*-value (F)	95% CI (G)	Total time saved (min) (H)
1	5	0.38 ± 0.25	0.38 ± 0.66	0	-	-	0.00
2	17	0.57 ± 0.25	0.56 ± 0.66	0.01 ± 0.71	9.47E-01	(-0.35, 0.38)	0.20
3	52	0.77 ± 0.25	0.75 ± 0.66	0.02 ± 0.71	8.47E-01	(-0.18, 0.22)	0.99
4	81	0.97 ± 0.25	0.91 ± 0.66	0.06 ± 0.71	4.78E-01	(-0.1, 0.21)	4.54
5	108	1.16 ± 0.25	1.08 ± 0.66	0.08 ± 0.71	2.29E-01	(-0.05, 0.22)	8.88
6	129	1.36 ± 0.25	1.27 ± 0.66	0.09 ± 0.71	1.30E-01	(-0.03, 0.22)	12.23
7	171	1.56 ± 0.25	1.44 ± 0.66	0.12 ± 0.71	2.91E-02	(0.01, 0.23)	20.32
8	140	1.76 ± 0.25	1.6 ± 0.66	0.16 ± 0.71	9.12E-03	(0.04, 0.28)	22.10
9	108	1.95 ± 0.25	1.74 ± 0.66	0.22 ± 0.71	1.81E-03	(0.08, 0.35)	23.48
10	89	2.15 ± 0.25	1.93 ± 0.66	0.22 ± 0.71	4.67E-03	(0.07, 0.37)	19.34
11	80	2.35 ± 0.25	2.08 ± 0.66	0.27 ± 0.71	1.04E-03	(0.11, 0.43)	21.51
12	43	2.55 ± 0.25	2.23 ± 0.66	0.31 ± 0.71	5.91E-03	(0.09, 0.53)	13.42
13	19	2.74 ± 0.25	2.5 ± 0.66	0.24 ± 0.71	1.57E-01	(-0.1, 0.58)	4.54
14	22	2.94 ± 0.25	2.67 ± 0.66	0.27 ± 0.71	8.79E-02	(-0.04, 0.58)	5.92
15	17	3.14 ± 0.25	2.88 ± 0.66	0.26 ± 0.71	1.55E-01	(-0.11, 0.62)	4.34
16	16	3.33 ± 0.25	2.94 ± 0.66	0.39 ± 0.71	4.02E-02	(0.02, 0.77)	6.31
17	16	3.53 ± 0.25	3.24 ± 0.66	0.3 ± 0.71	1.14E-01	(-0.08, 0.67)	4.74
18	18	3.73 ± 0.25	3.33 ± 0.66	0.39 ± 0.71	2.93E-02	(0.04, 0.75)	7.10
19	13	3.93 ± 0.25	3.59 ± 0.66	0.33 ± 0.71	1.13E-01	(-0.09, 0.76)	4.34
20	4	4.12 ± 0.25	3.78 ± 0.66	0.35 ± 0.71	3.85E-01	(-0.78, 1.47)	1.38
8.19 ± 3.65	1148	1.79 ± 0.25	1.63 ± 0.66	0.16 ± 0.71	1.88E-14	(0.12, 0.2)	185.66

The number of original prescription items (A) with corresponding number of items dispensed (B), time for dispensing prior to reduction (C), time for dispensing after reduction (D), reduction in time for dispensing (E), *p*-value for time saved (E), 95% confidence interval of time saved (F), and total time saved (H) for each of the original numbers of prescription items. The last row shows the average for all 1,148 prescriptions.

### Total cost savings

The total cost savings involves two issues. The first of which is the time saved due to item reduction (Table 3). The second issue is reduced powder loss during the preparation process. Based on data from our TCM pharmacy, one pharmacist dispenses on average 1,408 prescriptions of CCHE per month. By applying the CIPS algorithm, the mean time saved for each dispensing procedure is 0.16 min. Multiplied by the mean number of prescriptions, approximately 3.75 h could be saved each month. Assuming the average monthly salary for a pharmacist is $55,000 NTD, and the pharmacist works an average of 40 h per week, a 3.75-h reduction in dispensing time would translate to a reduction of $1,290 NTD per month per pharmacist. Thus, the annual labor savings for a given pharmacy would be $15,488 NTD per pharmacist.

In terms of powder loss during the dispensing process, the estimated loss of each item equates to approximately 0.13 g, while the average cost is $$\$2.62\pm 1.02$$ NTD/item/g. Thus, reducing one item from a prescription would save $0.33 $$\pm 0.13$$ NTD. As shown in Table 2, an average of 0.82 $$\pm$$ 1.01 items are reduced from each prescription, which means that $$\$0.28\pm 0.36$$ NTD would be saved by applying CIPS. With an average of 1,408 prescriptions dispensed each month per pharmacist, the cost incurred due to powder loss could be reduced by $376 NTD per month, or $4,517 NTD per year (*p* $$\le {10}^{-6}$$). If one considers the entire CMUH TCM pharmacy, the above numbers would be multiplied by 14.6 (246,749 CCHE prescriptions were dispensed in 2019 at our pharmacy), amounting to a total savings of $292,148 NTD per year for CMUH. Considering all TCM services in Taiwan, this would translate to a total savings of approximately $77 million NTD per year, equal to 0.3% of the entire National Health Insurance budget allocated to TCM. A summary of the cost savings is shown in Table 4.

**Table 4:** Estimated annual savings for an average pharmacist, at the CMUH TCM department, and for all TCM clinics/hospitals in Taiwan

	Annual savings for an average pharmacist	Annual savings for the CMUH TCM department	Annual savings for all TCM clinics/ hospitals (3839 in total)
Manpower savings	15,488 NTD (553 USD^a^)	226,187 NTD (8,078 USD^a^)	59 million NTD (2.1 million USD^a^)
Savings from reduced powder spray	4,517 NTD (161 USD^a^)	65,961 NTD (2,356 USD^a^)	17 million NTD (0.6 million USD^a^)
Total savings	20,005 NTD (714 USD^a^)	292,148 NTD (10,434 USD^a^)	77 million NTD (2.7 million USD^a^)

^a^USD were converted from NTD using an exchange rate of 28 NTD = 1 USD

### Feedback for CIPS by TCM pharmacists

Apart from the quantitative analysis included in our study, we also interviewed 5 TCM pharmacists regarding their opinions of CIPS and the effect on their work. Most of the pharmacists agreed that after applying the system, both the workload and powder spray could be expected to decrease, which is consistent with our quantitative findings. However, several pharmacists raised concerns about the increased difficulty of double checking whether there was a prescription error by the physician. This is because most physicians have their specific style of prescribing CCHE. When the physicians’ prescription which is forwarded to the pharmacist deviates from the norm, the pharmacist would be able to sense a possible error. However, when CIPS is applied, the physicians’ prescription pattern may be altered, thereby make it harder for the pharmacist to detect possible errors. Details of the pharmacists’ responses to questions are included in supplementary Table 1.

## Discussion

The ingestion of CCHE is the most common method for taking TCM in Taiwan. Single herbs or formulas are first boiled in water. The pieces of herbs and impurities are then filtered out. The resulting decoction of single herbs or formulas is subsequently concentrated into dried granules [7].

There are several advantages to these concentrated extracts. First, all CCHE are produced by GMP manufacturers in Taiwan, which assures the quality, consistency, and safety of the ingredients. Second, as the granules are concentrated, the dose can be easily adjusted according to individual requirements and can be consumed quickly and conveniently. Third, granules require significantly less storage space as compared to medicinal herbs. Additionally, the heating process eliminates insects and mold, which may contaminate the raw TCM herbal ingredients, thereby facilitating convenient storage and enhancing safety [8].

Concentrated extracts nevertheless have several disadvantages. TCM practitioners must convert the proportions when writing prescriptions, which may be quite different from the dosages offered in textbooks, and which may be inconvenient in a clinical setting. In addition, it becomes more difficult for practitioners to adjust the proportions when they use CCHE of compound formulas. This has led to a new way of prescribing Chinese herbal medicine, known in the field as “formula stacking". Most TCM practitioners now consider a compound formula as a single unit, and may thus include several concentrated extracts of compound formulas in a single prescription, as demonstrated in Table 1. Such a prescription may contain a wide variety of single herbs with multiple redundancies. In our example shown in Table 1, the four compound formulas each contain between 5 and 10 single herbs; however, there are many redundancies among the compounds. Indeed, of the 26 single herbs listed as components of the compounds, there are actually only 18 different single herbs. Since having more items to dispense equates to more time spent, redundancies naturally become obstacles to clinical efficiency. In addition, the lack of clarity regarding the exact herbal content of prescriptions may negatively affect the precision and efficacy of the prescriptions. Furthermore, as the CCHE granules have lost the physical traits of their original herbs, neither physicians nor pharmacists can visually inspect the quality of the products. Therefore, the quality control of the source materials must rely on government supervision and the manufacturer’s own ethical standards.

When developing CIPS, we focused on addressing some of the disadvantages inherent to CCHE. Our system can perform accurate calculations to reconvert compound formulas into the original combination of single herbs, thereby simplifying and improving the accuracy of prescriptions. By inputting real world prescription data into our program, a 9.31% reduction in the number of items can be achieved. This translates into an approximate 3.75-h reduction in work time for an average pharmacist per month. In terms of economic benefits, the process of reducing the medicinal components effectively lowers both the labor costs and drug loss involved in the prescription preparation process. We estimate that $20,005 NTD could be saved per year per pharmacist by implementing CIPS; moreover, this would be a total savings of $77 million NTD per year if implemented in all of Taiwan’s TCM clinical services. 

Despite demonstrating significant efficiencies and cost savings, this study has revealed limitations related to the CIPS algorithm. First, our recombination algorithm does not account for the exact dose prescribed by the physicians, which could decrease the percentage of item reduction we calculate in the example here. Second, our algorithm does not predict the potential effects of herbal combinations. However, studies have shown that interactions between herbs do exist [9–14], thus recombining herbs in different ways may alter the intended function.

## Conclusion

In this study, we used real-world data from the CMUH TCM pharmacy to analyze the effectiveness of CIPS. We found that both labor costs and drug losses incurred during the prescription preparation process can be reduced. In addition, the system can facilitate more precise CCHE prescriptions by avoiding herbal redundancies. Thus, implementing CIPS in a clinical setting could assist TCM practitioners and pharmacists to offer patients precise CCHE prescriptions within a simplified dispensing environment while reducing medical waste and labor costs thereby benefiting clinics, patients, and the National Health Insurance system.

## Supplementary Information

**Additional file 1.**

**Additional file 2: Supplementary Table 1.** Transcript of the interview with TCM pharmacists regarding CIPS.

## Data Availability

The datasets generated for this study are available on request to the corresponding authors.

## Abbreviations

TCM: Traditional Chinese Medicine
MOHW: Ministry of Health and Welfare
OPD: Outpatient department
CHM: Chinese herbal medicine
CCHE: Concentrated Chinese herbal extracts
CIPS: Chinese Intelligence Prescription System
CMUH: China Medical University Hospital
NTD: New Taiwan Dollars
