# Supplementary material for: Chinese Intelligence Prescription System improves prescription accuracy while decreasing labor and drug costs
Source: BMC Health Serv Res. 2023 May 22;23:514. doi: 10.1186/s12913-023-09487-4 (PMC10201794; doi:10.1186/s12913-023-09487-4)
Supplement: Supplementary file 2 — Additional file 2: Supplementary Table 1. Transcript of the interview with TCM pharmacists regarding CIPS. [file 12913_2023_9487_MOESM2_ESM.docx]

**Supplementary Table 1** Transcript of the interview with TCM pharmacists regarding CIPS

| Question 1: What are your opinions regarding the effects of CIPS on the dispensing of CCHE? Please list both advantages and disadvantages. | |
| --- | --- |
| Pharmacist 1 | If the number of prescription items is reduced, the dispensing process can be accelerated while the accuracy of each prescription can be easily assessed; although, CIPS should present a comparison of before and after modifications. |
| Pharmacist 2 | 1. Reduce the need for contact due to prescription error.  2. Reduce the time to review prescriptions.  3. Reduce the frequency of dispensing errors. |
| Pharmacist 3 | Advantages: The system is used to streamline prescriptions. If the goal is to reduce the number of prescription items, the number of prescriptions dispensed will thus be reduced.  Disadvantages: Uncertain logic of system designation will be less acceptable; additionally, the workflow should be smooth, otherwise it will increase the burden due to more action required. |
| Pharmacist 4 | If the pharmacist does not deliberately compare changes made by the system and only acts according to the provided results, the workflow will be improved. |
| Pharmacist 5 | Advantages: It can accurately restore the composition and dosages of single compounds in the prescription, helping pharmacists to effectively review the prescription.  Disadvantages: It is not easy to intuitively project backwards to the patient's health issues from the newly prescribed compounds. |

| Question 2: Do you expect the dispensing time to be reduced? | |
| --- | --- |
| Pharmacist 1 | The average time for pharmacists to dispense an item is about 18 seconds. Assuming 10 items per prescription, 10% reduction in number, and with about 800 prescriptions per day, we expect to save 240 minutes of dispensing time per day. |
| Pharmacist 2 | Fewer prescription items are expected to reduce the time to review prescriptions and dispense. |
| Pharmacist 3 | If the number of prescribed items by physicians who frequently provide prescriptions with a large number of items can be reduced, a “significant” reduction in pharmacist labor can be expected. |
| Pharmacist 4 | See response to Question 1. |
| Pharmacist 5 | Yes |

| Question 3: Do you expect reduced powder spray losses? | |
| --- | --- |
| Pharmacist 1 | Not necessarily, if the total amount is unchanged, the dose may simply be adjusted to other items. However, if the physician reduces the total prescribed dosage due to the reduction in item numbers, it is indeed possible to reduce the spray loss. |
| Pharmacist 2 | Reducing the number of prescription items can reduce the number of dispensed items, and thus reduce waste of powder. It can also reduce the potential health risks for the pharmacists. |
| Pharmacist 3 | It is possible to reduce waste under the current definition. |
| Pharmacist 4 | This generally depends on the dispensing habits of each pharmacist. But yes, it can be expected that reduced items would reduce powder spray losses. |
| Pharmacist 5 | Yes |

| Question 4: Do you expect increased work burden after the implementation of such a system? | |
| --- | --- |
| Pharmacist 1 | No |
| Pharmacist 2 | There is currently no additional burden |
| Pharmacist 3 | After the introduction of the system, there may be additional operations in the transfer process, and whether it is a burden will depend on the actual implementation. |
| Pharmacist 4 | See response to Question 1. |
| Pharmacist 5 | Pharmacists would need to switch thinking between the actual prescription content and the composition of the prescription after modifications. |
